# Modeling Neurological Diseases With Human Brain Organoids

**DOI:** 10.3389/fnsyn.2018.00015

**Published:** 2018-06-08

**Authors:** Hansen Wang

**Affiliations:** Faculty of Medicine, University of Toronto, Toronto, ON, Canada

**Keywords:** brain organoids, pluripotent stem cells, neurological diseases, neurodevelopmental disorders, neurodegenerative diseases, iPSCs, autism, Alzheimer disease

## Abstract

The complexity and delicacy of human brain make it challenging to recapitulate its development, function and disorders. Brain organoids derived from human pluripotent stem cells (PSCs) provide a new tool to model both normal and pathological human brain, and greatly enhance our ability to study brain biology and diseases. Currently, human brain organoids are increasingly used in modeling neurological disorders and relative therapeutic discovery. This review article focuses on recent advances in human brain organoid system and its application in disease modeling. It also discusses the limitations and future perspective of human brain organoids in modeling neurological diseases.

## Introduction

The diseases of central nervous system (CNS) usually are of complex and diverse etiologies, and could be further complicated by variable genetic, epigenetic and environmental factors that differ among individuals. One of the major limitations in the study of these diseases is the lack of an *in vitro* system that faithfully recapitulates the complexity and delicacy of human brain. Since the invention of method to generate induced pluripotent stem cells (iPSCs) from somatic cells, tremendous progress has been made in developing protocols for differentiating human iPSCs into various cell types, including neural cells. The iPSCs derived from patients are now widely used in disease modeling (Takahashi and Yamanaka, 2006; Yu et al., 2007; Wang and Doering, 2012; Shi et al., 2017). However, traditional two-dimensional (2D) cultures are still limited in utilizing iPSCs to model disease processes, particularly with regard to neurological diseases.

The three-dimensional (3D) culture models are more physiologically relevant concerning the spatial organization of tissues, cell-cell and cell-matrix connections. It is now possible to differentiate pluripotent stem cells (PSCs), including both embryonic stem cells (ESCs) and iPSCs, into 3D organ-like tissues named organoids (Lancaster and Knoblich, 2014a; Clevers, 2016). In recent years, protocols that utilize PSCs to generate brain organoids have been developed (Kadoshima et al., 2013; Lancaster and Knoblich, 2014b; Dyer, 2016; Di Lullo and Kriegstein, 2017; Giandomenico and Lancaster, 2017; Li Y. et al., 2017; Sutcliffe and Lancaster, 2017). In addition, evidence indicating that these organoids can be produced from more fate-restricted neural stem cells is emerging (Clevers, 2016; Monzel et al., 2017). The potential for human brain organoids to provide a more dynamic and biological system for study of human brain biology and diseases has been generating huge interest and excitement in the research communities.

This review article will provide a general introduction of recent advancements in human brain organoid models and their application in modeling neurodevelopmental and neurodegenerative diseases. It will also highlight the impact of human brain organoid system on our understanding of neurobiology and diseases and discuss the current challenges and future perspectives for this model system.

## Generation of Human Brain Organoids

With the development of culture technology, human PSCs now can be differentiated into a range of neural identities in 3D cultures. Thus far, the *in vitro* 3D models of human brain which have been introduced include the serum-free floating culture of embryoid body (EB)—like aggregates with quick re-aggregation (SFEBq; Eiraku et al., 2008), cortical spheroids (Paşca et al., 2015), cerebral organoids (Lancaster et al., 2013), forebrain organoids (Kadoshima et al., 2013), and other brain region-specific organoids. Brain organoids are not just more heterogeneous and complex than the 2D neural rosettes; the 3D neuroepithelium are also more continuous, can develop different brain region identities and thus better recapitulate the complex interaction and connection among brain regions and structures. Ever since the generation of brain organoids was firstly reported, the field has experienced explosive growth and innovation over the last few years (Lancaster et al., 2013; Lancaster and Knoblich, 2014a; Di Lullo and Kriegstein, 2017; Giandomenico and Lancaster, 2017; Lancaster, 2018; Paşca, 2018).

A number of protocols have been developed to generate brain organoids from human PSCs. The PSCs cultured under appropriate conditions can grow into 3D organoids. As a typical protocol, the method for generating cerebral organoids, which depends on the intrinsic ability of PSCs to self-organize upon precisely timed manipulation of culture conditions without external growth factors or other molecules, has been well documented (Lancaster and Knoblich, 2014b; Sutcliffe and Lancaster, 2017). Briefly, PSCs *in vitro* are allowed to develop into aggregates, the so-called EBs, which display differentiation of three germ layers. Upon transfer of EBs to minimal media, ectoderm (the most outer layer) acquires more neural properties, and differentiates into neuroepithelium, which later is the source of neural progenitors. One key development here is to combine the floating 3D aggregate approach with Matrigel, the components of extracellular matrix (ECM), by embedding the growing EBs into Matrigel (Lancaster et al., 2013). Matrigel scaffold supports the self-organization of neuroepithelium in 3D context and induces the correct polarity signal to form the apicobasally polarized neuroepithelial bud. The neuroepithelium further develops into structures that are remarkably similar to developing human brain. The support by Matrigel seems to be critical for the building of *in vitro* brain structures. Later agitation provided by bioreactors in the floating cultures promotes the formation of much larger cerebral organoids with fluid-filled cavities that resemble ventricles. As there are no inductive signals, the organoids exhibit various brain region identities of CNS, which typically are hindbrain, midbrain, forebrain and even retinal tissues (Lancaster and Knoblich, 2014b; Camp et al., 2015; Sutcliffe and Lancaster, 2017).

Human brain organoids have been shown to exhibit other complex 3D structures, such as the defined proliferative zones homologous to the ventricular zone (VZ), the inner subventricular zone (iSVZ) and outer SVZ (oSVZ), resembling the multi-layer progenitor zone organization of human brain (Lancaster et al., 2013; Qian et al., 2016). A significant improvement is the generation of neurons with identities of neocortical layers (Kadoshima et al., 2013; Lancaster and Knoblich, 2014b), with diverse neuronal types of all six cortical layers observed in forebrain organoids (Qian et al., 2016). The brain organoids mimic many other features of brain development, such as the establishment of discrete brain regions, and both radial and tangential migration of cortical neuron populations. Since brain organoids are not patterned by externally added growth factors or morphogens, their development relies purely on self-organization. A recent study has successfully illustrated the patterning events driving the self-organization during the development and differentiation of cerebral organoids (Renner et al., 2017). Various distinct ventral and dorsal regions are present and many of these regions are interconnected in forebrain regions, indicating that a range of dorso-ventral identities could be generated in cerebral organoids; the organoids contain forebrain organizing centers that secret growth factors which may participate in dorso-ventral patterning. Moreover, timed generation of neurons with mature morphologies, along with subsequent generation of astrocytes and oligodendrocytes do exist in the organoids (Renner et al., 2017). This classical phenotypic study indicates that brain organoids can recapitulate the spatial and temporal patterning events that govern human brain development (Renner et al., 2017). As brain organoids can be maintained for extended periods, they might potentially model later events such as neuronal survival, maturation and degeneration beyond brain developmental stage (Lancaster and Knoblich, 2014b; Quadrato et al., 2017).

Notably, brain organoids do not just faithfully capture the structural and cytoarchitectural aspects of human brain, their overall epigenomic and transcriptional programs could closely mimic that of human brain (Luo et al., 2016; Xiang et al., 2017). The transcriptomes of cortical cells in organoids match well with those of apical progenitors, intermediate progenitors and cortical neurons in *in vivo* brain at different stages of differentiation. Both organoids and fetal cortices contain cells whose transcriptomes indicate that they are in the transition between stages, suggesting that differentiation is a continuous process (Camp et al., 2015). Brain organoids establish the epigenomic and transcriptional programs essential for brain development. Organoids recapitulate many epigenomic and transcriptional features of human fetal brain (Luo et al., 2016; Qian et al., 2016). Consistent with the findings from early-stage organoids, the cells in brain organoids developed over extended terms (more than 9 months) also display the transcriptional profiling that are closely related to their endogenous counterparts (Quadrato et al., 2017). The physiological properties of neurons and neuronal networks in human brain organoids have also been investigated (Lancaster et al., 2013; Paşca et al., 2015; Quadrato et al., 2017). Neurons in human cerebral organoids after 75 days in culture exhibit spontaneous Ca^2+^ surges, which respond well to glutamate, indicating the existence of electrically functional glutamatergic cells (Lancaster et al., 2013; Lancaster and Knoblich, 2014b). Interestingly, the glutamate release within brain organoids is measurable by biosensor (enzyme-modified microelectrodes; Nasr et al., 2018). Brain organoids can further develop in culture past early developmental stage, to allow not only the formation of cell diversity, but also the maturation of neurons. Functional synapses are evident in organoids after 6 months in culture (Paşca et al., 2015). Brain organoids in culture of over 9 months go through substantial neuronal maturation, including formation of dendritic spines and establishment of spontaneously active neuronal networks. The neurons within organoids react to light stimulation of photosensitive cells, suggesting that, beyond early brain development, brain organoids could be potentially used to study neural circuit dysfunctions related to neurological diseases (Quadrato et al., 2017). Notably, the existence of mature astrocytes was also confirmed by transcriptomic, proteomic and functional analysis in human brain organoids (Dezonne et al., 2017; Sloan et al., 2017). Importantly, the astrocytes from brain organoids are functionally identical to those isolated from adult human brain (Dezonne et al., 2017). The molecular, cellular and physiological similarities between human brain organoids and *in vivo* brain indicate that brain organoids could be potentially used to study human brain biology and disease.

The brain organoid protocols have been formulated to give rise to specific brain regions. Addition of patterning factors enables generation of organoids of distinct brain regional subtypes (Kelava and Lancaster, 2016a; Di Lullo and Kriegstein, 2017). Procedures for generation of human brain organoids of different brain regions, including cerebral cortex (Kadoshima et al., 2013; Lancaster et al., 2013; Xiang et al., 2017), cerebellum (Muguruma et al., 2015), midbrain (Jo et al., 2016; Qian et al., 2016, 2018; Monzel et al., 2017), forebrain (Qian et al., 2016, 2018), hypothalamus (Qian et al., 2016, 2018), hippocampus (Sakaguchi et al., 2015) and even the medial ganglionic eminence (MGE; Bagley et al., 2017; Xiang et al., 2017), a critical ventral brain domain producing cortical interneurons, have been described. These organoids provide evidence that human PSCs can differentiate into different cell types and self-organize into specific 3D structures, thus recapitulating the main features of human brain. Brain region-specific organoids would be particularly important for the study of neurological conditions where certain brain regions are specifically or more severely affected.

The isolated regional identities do restrict the ability of brain organoids to recapitulate certain interaction and connectivity among brain regions and structures. A critical advancement in recent studies is that organoids of different brain regions can be fused in culture for analysis of neuron migration, neural connectivity and networks (Mich et al., 2017). The studies have developed coculture method to fuse organoids of two adjacent brain structures, the dorsal (excitatory) and ventral (inhibitory) forebrains, within one organoid tissue (Bagley et al., 2017; Birey et al., 2017; Xiang et al., 2017). When the dorsal and ventral forebrain organoids are fused, a dorsal–ventral axis is formed and the GABAergic interneuron migration from ventral to dorsal forebrain resembling that of *in vivo* cortical interneurons could be observed by time-lapse imaging, demonstrating that cerebral organoid fusion cultures could be potentially applied to model complicated interaction between different brain regions (Bagley et al., 2017). Similarly, Xiang et al have successfully generated human PSC-derived MGE- and cortex-specific organoids that recapitulate the development of their *in vivo* counterparts and produce functional neurons and neuronal connections. After fusing the MGE- and cortex-specific organoids, human interneuron migration and integration could be visualized by live imaging (Xiang et al., 2017). Interestingly, migration of interneurons could be blocked by inhibition of CXCR4 (Bagley et al., 2017) and myosin II (Xiang et al., 2017), suggesting that the migration requires these molecules that are known to be involved in *in vivo* interneuron migration. Importantly, those migrated inhibitory neurons are physiologically active and could be synaptically integrated with excitatory neurons (Xiang et al., 2017). These studies thus represent a significant development in the brain organoid technology. The organoid fusion technology might offer researchers new opportunity to study brain cellular dynamics and neural circuitry and could be applicable for neurological disease modeling.

## Modeling Neurological Diseases With Organoids

As the species differences limit the resemblance of animal models to human biology, human brain organoids are particularly suitable for addressing biological issues or questions that would benefit from human model systems. For instance, brain organoids have been used to examine cell division orientation in human radial glial cells (Lancaster et al., 2013) and human cortical progenitor expansion (Otani et al., 2016), processes that might be uniquely regulated in humans compared to other species. Although animal models have been a powerful tool for understanding the roles of identified mutated genes in diseases, many of cognitive or psychiatric disorders, such as autism and schizophrenia, are of polygenic etiology and difficult to study with currently available animal models. Human brain organoids are representing an unprecedented opportunity to model complex polygenic neurological disorders, including those with unidentified genetic defects, and to study epigenetic changes that connect genes and environment underlying neurobiology of diseases (Luo et al., 2016; Forsberg et al., 2018; Janssens et al., 2018).

**Figure 1:**
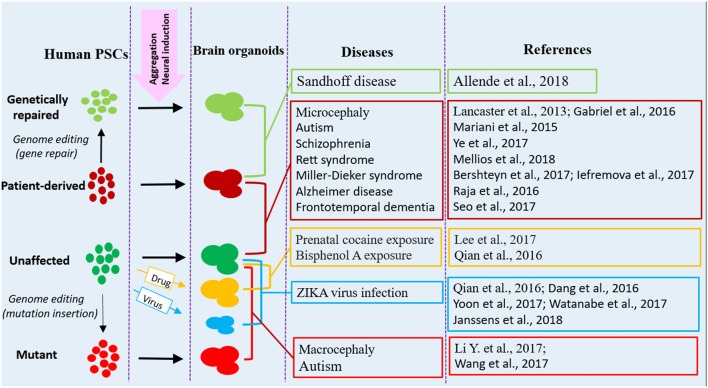
Human brain organoid modeling of neurological diseases. The brain organoid models could be generated using iPSCs derived from patients or by inflicting healthy brain organoids with drugs or virus when the organoids are formed. Alternatively, gene mutations could be introduced into unaffected human PSCs by genome editing (CRISPR-Cas9) to generate brain organoids with relative genetic defects. Patient brain organoids can also be genetically repaired to create isogenic controls. The diseases and references listed here are representative, but unexhausted. Abbreviations: iPSCs, induced pluripotent stem cells; PSCs, pluripotent stem cells.

**Figure 2:**
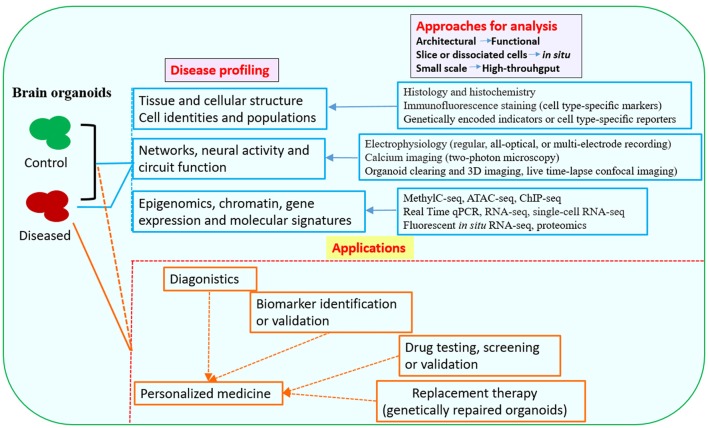
Downstream analysis and applications of human brain organoid models of neurological diseases. Conventional methods used in neurobiology might not be able to exploit the neuronal activity and network connectivity within the 3D architecture of brain organoids. The combination of advanced multi-level and high-throughput detection techniques which allow analysis of the whole or intact brain organoids, will greatly benefit phenotypic profiling of human brain organoid models and may help to further uncover mechanisms for disease pathogenesis (Mariani et al., 2015; Luo et al., 2016; Qian et al., 2016; Quadrato et al., 2016; Bershteyn et al., 2017; Hartley and Brennand, 2017; Renner et al., 2017; Xiang et al., 2017; Paşca, 2018). The brain organoid models could be potentially used in drug testing or screening, identification of new biomarkers or development of innovative diagnostics and therapeutics. Patient-derived brain organoids, together with drug testing, diagnostic or therapeutic approaches, might eventually lead to personalized medicine for the individuals (Kelava and Lancaster, 2016a; Qian et al., 2016; Quadrato et al., 2016; Di Lullo and Kriegstein, 2017; Quadrato and Arlotta, 2017). Abbreviations: ATAC-seq, assay for transposase-accessible chromatin with high-throughput sequencing; ChIP-seq, chromatin immunoprecipitation and sequencing; MethylC-seq, MethylC-sequencing; qPCR, quantitative PCR; RNA-seq, RNA-sequencing.

The brain organoid system has numerous advantages over animal models, holding the promise for studying at least some key features of diseases at 3D *in vitro* conditions. Organoid models are particularly useful for studies that need live and functional tissues, such as electrophysiological analysis and live imaging of dynamic cell behaviors. In addition, the potential for using brain organoid models are expanding with the development of genome editing tools, which allow for precisely targeted mutations or targeted gene repairs, thus spurring a further interest in brain organoid techniques. Human brain organoids are providing a new tool to investigate the mechanisms of neurological diseases that otherwise might be impossible to examine in animal models (Di Lullo and Kriegstein, 2017; Paşca, 2018). Currently, a variety of neurological disorders can be modeled with human brain organoids; various strategies and approaches have been utilized in disease modeling, downstream analysis and application (Figures 1, 2).

### Microcephaly

Microcephaly is a condition where patients with congenital diseases have significantly reduced brain size for their age. Mutations in the *CDK5RAP2* gene could affect the neocortical progenitor cells and have been reported in patients with microcephaly (Bond et al., 2005; Buchman et al., 2010). However, mouse models failed to recapitulate the symptoms of microcephaly in humans. Interestingly, cerebral organoids derived from a microcephaly patient with a truncating mutation in *CDK5RAP2*, exhibited premature neural differentiation, a defect that reflects the disease phenotype (Lancaster et al., 2013). Consistently, CDK5RAP2 levels are at least in part responsible for the observed hypoplasia, as RNA interference (RNAi) in control organoids was able to reproduce the phenotype, while CDK5RAP2 overexpression was able to partially rescue it (Lancaster et al., 2013). The study firstly demonstrates that brain organoid system is applicable for disease modeling. Seckel syndrome with microcephaly is caused by mutation of *centrosomal-P4.1-associated protein* (*CPAP*), which might reduce the population of neural progenitor cells during brain development (McIntyre et al., 2012; Garcez et al., 2015). Gabriel et al. (2016) reported that CPAP could negatively regulate the length of cilium, which plays a role in the maintenance of neural progenitor cells, in both 2D cultures and 3D organoids. While CPAP normally provides a scaffold for the cilium disassembly complex (CDC), the mutated one fails to do so, and results in inefficient recruitment of CDC, retardation of cilium disassembly, long cilia and delayed cell cycle re-entry, leading to premature differentiation of neural progenitor cells derived from patient iPSCs. Importantly, aberrant CDC function was confirmed to account for premature differentiation of neural progenitor cells in brain organoids of Seckel syndrome patient (Gabriel et al., 2016). This study reveals a novel mechanism for microcephaly caused by mutation of *CPAP*. The ability of human brain organoids to recapitulate brain development and microcephaly with high fidelity is further supported by a recent study in modeling microcephaly caused by mutation of *Aspm* gene (Li R. et al., 2017).

### Macrocephaly

Human brain organoids have also been used to model macrocephaly. As growth factors, the phosphatase and tensin homolog (PTEN)-protein kinase B (PKB/AKT) cascade regulates human cortical formation (Lee et al., 2012; Mirzaa et al., 2013). PTEN heterozygous loss-of-function mutations have been found in patients with macrocephaly (Butler et al., 2005; Marchese et al., 2014). In their cerebral organoid system, Li Y. et al. (2017) found that deletion of *PTEN* gene by CRISPR/Cas9-mediated genome editing increases AKT activity in human neural progenitors, promotes cell cycle re-entry, and transiently delays neuronal differentiation, resulting in a pronounced expansion of the radial glia and intermediate progenitor cells. Both human and mouse organoids that lack PTEN show a significant increase in size, but only human organoids display substantial surface folding (Li Y. et al., 2017). The phenotypic differences here might reflect inherent species differences in signaling regulation, cellular response, or anatomical organization. This study thus provides new insights into the mechanisms regulating the development of human cortex. It supports that human brain organoids can be used to study human specific phenotypes and underlying mechanisms.

### Autism and Other Psychiatric Disorders

Autism spectrum disorders (ASDs) are a group of neurodevelopmental and psychiatric conditions characterized by language deficits, social communication difficulties and repetitive behaviors, which are present in patients at their childhood (Wang and Doering, 2015; Mullins et al., 2016; Sztainberg and Zoghbi, 2016). Human brain organoids recapitulate early brain developmental events and could be invaluable for studying the neurobiology of ASDs. When compared with their control, cerebral organoids derived from patients with idiopathic autism displayed abnormal proliferation of neural progenitor cells and an increased population of inhibitory GABAergic neurons (Mariani et al., 2015), supporting the hypothesis about excitatory/inhibitory imbalance in the autistic brain (Rubenstein, 2010). Transcriptomic analysis showed that the transcription factor FOXG1 is upregulated in ASD organoids. Overexpression of FOXG1 was confirmed to be responsible for the overproduction of GABAergic neurons by using RNAi, suggesting that the shifting toward GABAergic neuron fate due to upregulation of FOXG1 could be a potential molecular mechanism in ASDs (Mariani et al., 2015). This study indicates that altered proliferation/neurogenesis equilibrium during early brain development might be a main attribute in at least a subgroup of ASDs.

The *Chromodomain helicase DNA-binding protein 8* (*CHD8*), a causal gene of ASDs, encodes a member of the CHD family of ATP-dependent chromatin-remodeling factors (Krumm et al., 2014). Mutations of this gene have also been associated with intellectual disabilities and schizophrenia (McCarthy et al., 2014). To investigate the function of CHD8 in human brain, *CHD8*^+/−^ cerebral organoids were created by CRISPR-Cas9 gene editing of iPSCs (Wang et al., 2017). In an extensive transcriptome analysis of CHD8 target genes in *CHD8*^+/−^ cerebral organoids and their isogenic controls, the identified differentially expressed genes (DEGs) were not only consistent with many of those found in the analysis in neural progenitor cells and neuronal cultures, but also significantly overlapped with the DEGs previously identified in idiopathic ASDs (Wang et al., 2017). Interestingly, DLX6-AS1, a long non-coding antisense RNA which regulates the development of GABAergic interneurons, was a top DEG in both datasets (Wang et al., 2017). The shared downstream signaling molecule DLX6-AS1 among different autism-causing genetic variants might be particularly important in designing potential therapeutics for ASDs.

Mutations of disrupted-in-schizophrenia 1 (DISC1) are known to be associated with major psychiatric disorders, such as schizophrenia, autism, bipolar disorder, and depression (Porteous et al., 2011; Narayan et al., 2013; Prytkova and Brennand, 2017). Although hundreds of DISC1-binding proteins have been identified, little is known about how DISC1 interacts with those proteins to affect human brain development (Porteous et al., 2011; Bradshaw and Porteous, 2012; Lipina and Roder, 2014). With insights from the structure of DISC1 in complex with its binding domain of Ndel1 as reported in their recent article, Ye et al. (2017) elegantly demonstrated that DISC1 can regulate kinetochore attachment of Ndel1, but not its centrosome localization during mitosis; disrupting DISC1/Ndel1 complex formation prolongs mitosis, influences cell-cycle progression in human cells, and causes deficits in cell-cycle of radial glial cells in both mouse embryonic cortex and human forebrain organoids. More interestingly, these deficits could be confirmed in organoids derived from a schizophrenia patient with DISC1 mutation that disrupts its interaction with Ndel1 (Ye et al., 2017). This study thus uncovers a new mechanism for DISC1 as a major hub protein at the crossroads of neurodevelopment, neuronal signaling and psychiatric disorders.

### Rett Syndrome

Rett syndrome (RTT) is an X-linked early-onset neurodevelopmental disorder that is predominantly caused by mutations of the* methyl-CpG-binding protein 2* (*MECP2*) gene, which encodes a multifunctional epigenetic regulator (Amir et al., 1999; Castro et al., 2013). The function of MeCP2 has been intensively studied in different models. However, its role in early brain development is poorly investigated. As microRNAs (miRNAs) play important roles in neurogenesis and are known to be downstream targets of MeCP2 (Szulwach et al., 2010; Wu et al., 2010), Mellios et al. (2018b) employed RTT patient-derived iPSCs and MeCP2 RNAi approaches to identify novel MeCP2-targeted miRNAs during neuronal development. They found that miR-199 and miR-214 are upregulated during early brain development and differentially modulate extracellular signal regulated kinase (ERK)/mitogen-activated protein kinase (MAPK) and AKT signaling pathway. Meanwhile, defects in neurogenesis, neuronal differentiation and migration were revealed in both MeCP2-deficient and patient-derived cerebral organoids (Mellios et al., 2018a,b). Inhibition of miR-199 or miR-214 expression in MeCP2-deficient neural progenitor cells rescued the related signaling pathway and ameliorated the alterations in neuronal differentiation (Mellios et al., 2018b). This study thus depicts a novel signaling pathway mediated by MeCP2-targeted miRNAs that might influence neurogenesis and early brain development.

### Miller-Dieker Syndrome

Miller-Dieker syndrome (MDS), a congenital form of lissencephaly, is caused by a heterozygous deletion of chromosome 17p13.3 leading to malformations during cortical development (Matarese and Renaud, 2009; Nagamani et al., 2009). Previous information about this disease largely came from limited analysis of postmortem human brains and mouse models. However, the mouse models have disadvantage of being naturally lissencephalic. Thus, human brain organoids are recently used to investigate how MDS affects human progenitor subtypes (outer radial glia cells) that control neuronal output and influence brain topology. By analyzing cerebral organoids derived from control and MDS-iPSCs, Bershteyn et al. (2017) observed a cell migration defect that could be rescued by correction of the chromosomal deletion, and severe apoptosis of the founder neuroepithelial stem cells, accompanied by increased horizontal cell divisions. They also identified a mitotic defect in outer radial glia, a progenitor subtype that is largely absent from lissencephalic rodents but critical for human neocortical expansion (Bershteyn et al., 2017). Similarly, Iefremova et al. (2017) used patient-specific forebrain organoids to investigate pathological changes associated with MDS. They found that patient-derived organoids are reduced in size, a change accompanied by a switch from symmetric to asymmetric cell division of VZ radial glia cells; the N-cadherin/β-catenin signaling axis is disturbed; restoring active β-catenin signaling rescues division modes and ameliorates growth defects in the organoids (Iefremova et al., 2017). Although human brain organoids might not well recapitulate gyrification, they might have relevant cell types, cellular or molecular mechanisms necessary for the disease formation. The studies, therefore, provide insight into cellular pathogenesis of lissencephaly and broaden the utility of brain organoids for modeling human neurodevelopmental disorders.

### Sandhoff Disease

Sandhoff disease is an autosomal recessive condition caused by mutation in beta-hexosaminidase β subunit (HEXB) which results in the loss of beta-hexosaminidase activity and subsequent lysosomal accumulation of its substrate, GM2 ganglioside (Cordeiro et al., 2000; Ferreira and Gahl, 2017). This lysosomal storage disorder is characterized by catastrophic neuropathies and death at early childhood. However, it is largely unknown how lysosomal accumulation of ganglioside could influence brain development. To generate the neurodevelopmental model for Sandhoff disease, Allende et al. (2018) developed cerebral organoids with iPSCs derived from the fibroblasts of an infant with Sandhoff disease; they also created the isogenic (HEXB-corrected) controls by genome editing using CRISPR/Cas9 technology. As expected, the Sandhoff disease organoids, but not the control organoids, displayed accumulated GM2 ganglioside. The diseased organoids also exhibited increased size and cellular proliferation compared to their isogenic controls. As indicated by transcriptomic analysis, there was impairment in the development of those Sandhoff disease organoids (Allende et al., 2018). This study suggests that altered neuronal differentiation might be an early developmental event in Sandhoff disease.

### Prenatal Drug Exposure

Previous studies have shown that prenatal cocaine exposure to rats induces cytoarchitectural and related signaling changes in the embryonic neocortex (Lee et al., 2008, 2011). However, the species differences in brain development and metabolism limit the translation of these findings to humans. Human brain organoids are offering more relevant models to study the influence of prenatal drug exposure on human fetal brain. Using the neocortical organoids, Lee et al. (2017) demonstrated that cocaine exposure could inhibit proliferation of neocortical progenitor cells, cause premature neuronal differentiation and interrupt the development of neural tissues. These effects are likely mediated by CYP3A5-induced generation of reactive oxygen species as knockdown of CYP3A5 reversed cocaine-induced pathologies in the brain organoids, suggesting that CYP3A5 might be a therapeutic target for treatment of neurodevelopmental disorders related to prenatal cocaine exposure (Lee et al., 2017). The study also indicates that human brain organoids provides a platform for investigating effects of abused substances or drugs on CNS.

### ZIKA Virus Infection

ZIKA virus (ZIKV) is a pathogen causing congenital microcephaly and neurological disorders. With its outbreak of in the Americas, human brain organoids are becoming a powerful tool for studying the effects of ZIKV on brain development, helping to establish the causal relationship between ZIKV infection and the selectively targeted destruction of neural progenitor cells (Garcez et al., 2016; Ming et al., 2016). ZIKV has been found to directly infect cortical neural progenitors in different experimental models, including human iPSC-derived neural progenitors in monolayer, 3D neurospheres and brain organoids (Garcez et al., 2016; Li et al., 2016; Ming et al., 2016; Qian et al., 2017). Studies in forebrain organoids revealed the cell-specific viral tropism; namely, ZIKV selectively affects neural progenitor cell proliferation (Qian et al., 2016). Productive infection of neural progenitor cells by ZIKV delays cell cycle progression and increases cell death, causing a reduction in organoid size resembling microcephaly (Dang et al., 2016; Ming et al., 2016). At the molecular level, ZIKV infection results in dysregulation of multiple signaling pathways (Wen et al., 2017). The innate immune receptor Toll-like receptor 3 (TLR3) is upregulated after ZIKV infection; inhibition of TLR3 partially rescued the phenotypic effect of ZIKV in human brain organoids (Dang et al., 2016). Further analysis of gene expression during TLR3 activation identified genes related to neuronal development, suggesting the molecular mechanisms underlying disrupted neurogenesis (Dang et al., 2016). Interestingly, a recent study has demonstrated that ZIKV infection might just cause subtle (epigenetic) changes in brain, as the infection of brain organoids alters DNA methylation in multiple cell types (neural progenitors, astrocytes and neurons) at genes that are associated with various neuropsychiatric disorders, suggesting that prenatal ZIKV infection might potentially cause brain diseases later in life (Janssens et al., 2018).

How ZIKV can directly interact with the cells to influence neurogenesis? The ZIKV genome consists of a positive-sense, single-stranded RNA, which encodes a single open reading frame (ORF; White et al., 2016). Translation of the ORF generates a large polyprotein of over 3000 amino acid residues, which can be further cleaved to produce three structural proteins (C, prM, and E) and seven non-structural proteins (NS1, NS2A, NS2B, NS3, NS4A, NS4B and NS5; White et al., 2016). ZIIKV-NS4A and NS4B have been found to inhibit the AKT-mammalian target of rapamycin (mTOR) signaling pathway, resulting in defects in neurogenesis and abnormal activation of autophagy (Liang et al., 2016). A recent study showed that ZIKV-NS2A, but not Dengue virus (DENV)-NS2A, causes reduced proliferation and premature differentiation of radial glial cells and aberrant positioning of newborn neurons. Mechanistically, ZIKA-NS2A, but not DENV-NS2A, interacts with adherens junction (AJ) complex and destabilizes the complex, leading to impairment of AJ complex formation and aberrance of radial glial fiber scaffolding in embryonic mouse cortex. Similarly, ZIKA-NS2A, but not DENV-NS2A, reduces proliferation of radial glial cells and causes deficits in AJ in human forebrain organoids (Yoon et al., 2017). These studies thus reveal the molecular and cellular components involved in ZIKV infection during mammalian brain development.

Few potential options are currently available to treat the devastating ZIKV infection. Chemical-based drugs might become a first-response option. Human PSC-derived neural progenitors and brain organoids are providing platforms for drug testing and validation. Zhou et al. (2017) reported a high-content chemical screen using human PSC-derived cortical neural progenitor cells. They found that hippeastrine hydrobromide (HH) and amodiaquine dihydrochloride dehydrate (AQ) can inhibit ZIKV infection in neural progenitor cells. Further validation showed that HH also rescues ZIKV-induced growth and differentiation defects in neural progenitor cells and human fetal-like forebrain organoids. Additionally, HH and AQ inhibit ZIKV infection in adult mouse brain *in vivo*; HH also suppresses viral propagation in adult mice with active ZIKV infection, highlighting its therapeutic potential. However, AQ exhibits high cytotoxicity in brain organoid cultures, raising safety concerns if the drug is used during pregnancy (Zhou et al., 2017). Watanabe et al. (2017) developed enhanced organoid methods to model ZIKV-associated microcephaly; they identified more potential susceptibility receptors (other transmembrane receptors of the TAM/TIM family, including TYRO3, MER and TIM1) for ZIKV entry into neural progenitors and compounds (Ivermectin and Duramycin) that can mitigate ZIKV-induced cytopathy. Collectively, the studies demonstrate the power of brain organoids in helping to identify drug targets or candidates for further testing.

As the discrepancy exists in tissue architecture and cell type composition between human brain organoids and *in vivo* brain, the findings regarding cell type infectivity in brain organoids and their significance for human ZIKV infection need to be carefully interpreted. High infectivity of neural progenitor cells was found in both organoids and primary tissue. However, infection of astrocytes was only occasionally seen in organoids (Qian et al., 2016) but was abundantly present in primary tissue (Retallack et al., 2016). This could be due to either a lower percentage of astrocytes in brain organoids or inherent differences between *in vitro*- and *in vivo*-derived cells. The vulnerability of microglia to ZIKV infection was observed in primary tissues (Retallack et al., 2016), whereas this cell type is usually absent in brain organoids, which should be taken into account when investigating the viral entry mechanisms. AXL, also known as tyrosine-protein kinase receptor UFO, has been identified as a candidate viral entry receptor in human primary tissues and is highly expressed in radial glia cells of brain organoids (Nowakowski et al., 2016). However, genetic ablation of AXL had no effect on ZIKV entry or ZIKV-mediated cell death in human iPSC-derived neural progenitor cells or early-stage cerebral organoids (Wells et al., 2016). Fortunately, the apparent contradiction in these findings could be tentatively explained by another study documenting that AXL is essential for infection of human glial cells (astrocytes and microglia), but not for human neural progenitor cells (Meertens et al., 2017). The significance of AXL or other signaling molecules involved in glial ZIKV infection, and the susceptibility of glial cells to infection might not have been realized with brain organoid models alone, suggesting that optimization of brain organoids or combination with other model systems are required for better modeling human ZIKV infection (Di Lullo and Kriegstein, 2017).

### Neurodegenerative Diseases

The development of human iPSC-derived models for late-onset neurodegenerative diseases has been challenging due to the immaturity of neurons derived *in vitro*. Studies have suggested that brain organoids could be relevant models for neurodegenerative diseases such as Alzheimer disease (AD), the most common type of dementia, which is mainly characterized by extracellular deposition of misfolded amyloid-β (Aβ) containing plaques and intracellular neurofibrillary tangles (NFTs; Hardy and Selkoe, 2002; van der Zee et al., 2008; Querfurth and LaFerla, 2010). As 2D culture models do not have the complex extracellular environment necessary for extracellular protein aggregation, 3D culture conditions are more promising for modeling Aβ and Tau pathologies and testing of candidate drugs (Choi et al., 2014; Lee et al., 2016). Raja et al. (2016) have developed a scaffold-free culture method to generate brain organoids using iPSCs derived from patients with familial AD (FAD). These organoids could reproduce AD-like pathologies, including amyloid aggregation, hyperphosphorylated tau protein and abnormalities of endosomes. These pathologies could be age-dependent as observed in organoids of multiple patients. Furthermore, when those patient organoids were treated with β- and γ-secretase inhibitors, Aβ and tau pathology were significantly reduced (Raja et al., 2016). Notably, these findings are particularly significant as the phenotypes have never been reported in mouse models with FAD mutations. This study first used human brain organoids to investigate age-related neurodegeneration, demonstrating the potential of human brain organoids to increase the translatability of preclinical drug discovery in AD. The increase of p25, a proteolytic fragment of the regulatory subunit p35, aberrantly activates cyclin-dependent kinase 5 (Cdk5), which has been known to be involved in neurodegenerative disorders, including AD. p25/Cdk5 plays roles in tauopathy, as a recent study showed that blocking p25 generation by replacing endogenous p35 with the non-cleavable mutant p35 (Deltap35) reduced phosphorylation of tau and its seeding activity in the brain of mice overexpressing mutant human tau (P301S) and thus attenuated synaptic loss and LTP impairment at hippocampal CA3 region of those mice (Seo et al., 2017). In validating the role of p25/Cdk5 in tauopathy, the authors derived iPSCs from frontotemporal dementia patient with Tau P301L mutation, generated P301L:Deltap35KI isogenic iPSC lines using CRISPR/Cas9 genome editing, and further created cerebral organoids from the isogenic iPSCs. They found that blockade of p25 generation reduced tau phosphorylation and increased the level of synaptic marker synaptophysin in the organoids (Seo et al., 2017). The data demonstrate that p25/Cdk5 mediates tau-associated pathology. Human brain organoid models are important for validating findings from mouse models. Together, these studies indicate that human brain organoids are hopefully providing a therapeutic discovery platform for neurodegenerative diseases.

There are many other neurodegenerative diseases, like Parkinson disease and Huntington’s disease, which could be potentially studied using human brain organoids. The generation of functional human midbrain-like organoids (containing dopaminergic neurons) from human PSCs or more fate-restricted neural stem cells suggests that human brain organoids may ultimately be valuable for modeling age-related neurological diseases like Parkinson disease (Jo et al., 2016; Monzel et al., 2017).

**Figure 3:**
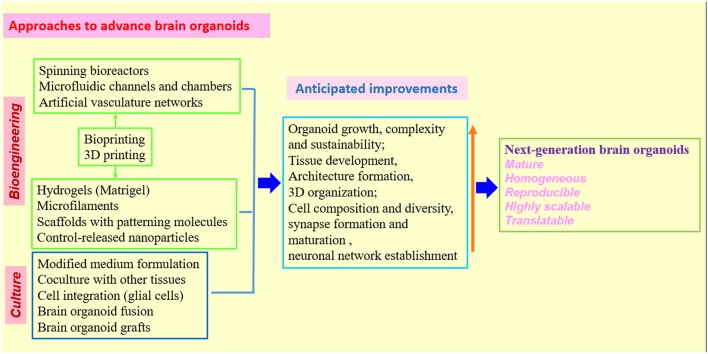
Strategies and approaches to optimize human brain organoid models. Combination of biomaterials, bioengineering approaches with improved 3D culture techniques will be needed to create the next generation of human brain organoid models (Kelava and Lancaster, 2016a,b; Qian et al., 2016; Yin et al., 2016; Bagley et al., 2017; Lancaster et al., 2017; Paşca, 2018). While standardized protocols and shared methodologies could be beneficial to research community, the combination of different organoid techniques must be rationalized in individual studies to better model human brain.

Although not every aspect of brain disease can be modeled by organoids, the earlier events in disease progression, which are more likely to be recapitulated and studied, may share molecular and cellular mechanisms with disease manifestations at later stages. Undoubtedly, rational approaches for phenotypic profiling of brain organoid models and for optimization of brain organoid cultures are needed (Figures 2, 3). Future studies will be able to build upon the afore-mentioned groundworks to advance brain organoid techniques for modeling both neurodevelopmental and neurodegenerative diseases and elucidating novel aspects of disease pathogenesis.

## Limitations, Challenges and Future Directions

While *in vitro* organoids developed without the presence of normal embryonic surrounding have the advantages for visualization and manipulation of cellular processes in the tissues, they lack the developmental and patterning cues, which are essential for organ development. Owning to the missing of surrounding supportive tissue and body axes, brain organoids do not organize themselves into the same shape or pattern of the *in vivo* brain although they do develop discrete brain regions (Lancaster et al., 2013; Kelava and Lancaster, 2016a). Axial patterning signals, which in turn affect the formation of different brain regions, could be applied to the organoids by mimicking endogenous developmental signaling gradients using controlled signal-releasing beads, or by culturing organoids on signaling molecules-coated scaffolds (Hartley and Brennand, 2017). Additionally, most protocols mainly depend on the ability of stem cells to self-organize into distinct brain structures. This spontaneous acquisition of cell identity can cause inconsistency in producing the desired tissues, resulting in heterogeneity or “batch-effects” that can cover real phenotypes if different batches of organoids are significantly variable in quality and brain regions they generate. Currently, there is an urgent need for homogeneous brain organoids to consistently reproduce relative phenotypes of neurological diseases (Quadrato et al., 2016). As the PSCs show significant variability which depends on the cell lines, passages, and even the properties of colonies or cells within a colony, careful selection of cell lines, and methods for reprogramming into iPSCs and culturing the cells should be considered in designing new organoid protocols. Meanwhile, the organoid field will benefit from standardized protocols and shared methodologies across different laboratories (Lancaster and Knoblich, 2014b; Kelava and Lancaster, 2016a,b; Prytkova and Brennand, 2017) as more different organoid protocols will be developed and coexist to provide options for studies depending on the strength of a specific protocol and the questions to address.

The absence of vascularization restricts the supply of oxygen and nutrients within organoids. Brain development, especially at the late stages, highly depends on the vascularization of SVZ, as being close to blood vessels is necessary for differentiation of neural progenitor cells and neuronal survival and maturation (Kelava and Lancaster, 2016a). The absence of vascularization is probably responsible for the shortage of progenitor populations and potentially making it difficult to replicate correct cortical plate formation in brain organoids; it also causes necrosis in the center of organoids when the volume of organoids expands beyond the limit of perfusion. These disadvantages greatly limit the developmental potential or maturation that can be modeled by the system (Giandomenico and Lancaster, 2017). To faithfully model *in vivo* brain, efforts have been focused on delivering oxygen, nutrients or signaling molecules deeper into the organoids, by culture modifications or engineering approaches. One option to overcome the absence of vascularization would be using bioreactors or microfluidics to help drive the flow of media and distribute nutrients to organoids. Such techniques help to generate larger and more mature organoids (Kelava and Lancaster, 2016a; Qian et al., 2016). Artificial tissues could be used to mimic vascular networks. Growing organoids directly on a microfluidic channel with endothelial cells or signaling molecule carriers to facilitate angiogenesis could become another option (Kelava and Lancaster, 2016b; Yin et al., 2016). Notably, combination of tissue-specific cells with mesenchymal cells to promote vascularization in transplantation of an organ bud might represent an option in refining protocols to generate vascularized organoids (Takebe et al., 2015). To further overcome the limitations of *in vitro* systems, a recent study developed a method to transplant human brain organoids into adult mouse brain (Mansour et al., 2018). The grafted organoids showed progressive differentiation and maturation of neurons, generation and integration of glial cells, and growth of axons to the host brain. Interestingly, functional intra-graft neuronal network activity and graft-to-host synaptic connectivity were formed, and blood vessels could be detected in the grafts (Mansour et al., 2018). This study thus provides an *in vivo* model of functional and vascularized brain organoids. Vascularized brain organoids would be a powerful tool to model the blood-brain barrier (BBB) and investigate drug transport through BBB. Importantly, vascularized organoids, which more precisely mimic *in vivo* brain anatomy and physiology, may facilitate brain disease modeling and also present an ideal platform for drug testing (Kelava and Lancaster, 2016a; Lancaster, 2018).

It seems that most of current organoid protocols or medium formulations favor progenitor cells and might not well support the formation of dendritic spines and mature synapses for neurons in brain organoids. Although neurons in brain organoids make functional synapses, much more need to be done to find out the conditions most suitable for the establishment of neural circuitry and neurophysiological activity (Kelava and Lancaster, 2016a). To improve the conditions in the organoid generation protocols, the derivation and/or integration of non-neuronal cells, such as astrocytes, oligodendrocytes, microglia, meningeal cells or cells of the vasculature, will need to be considered. The presence of astrocytes and oligodendrocytes in cerebral organoids is critical given the roles that the cells play in synaptogenesis, myelination, circuit maturation and disease initiation/progression (Qian et al., 2016; Hartley and Brennand, 2017; Micu et al., 2018; Paşca, 2018). Microglia, the resident innate immune cells of the CNS, are actively involved in neuronal development, maturation and homeostasis. Due to the developmental origin of microglia outside of CNS, microglia are absent from human PSC-derived brain organoids and the integration of microglia or their precursors into the organoids will be a challenge (Muffat et al., 2016a). Optionally, PSC-derived cells of different types could be differentiated separately and later integrated into the organoids. The efficient generation of microglia from human PSCs may indicate a potential to incorporate microglia into brain organoids during the development of those organoids (Muffat et al., 2016b; Pandya et al., 2017). Interestingly, human PSC-derived neural progenitors, mesenchymal stem cells, endothelial cells, and microglia precursors could be combined on chemically engineered hydrogels to form 3D neural cultures with microglia and vascular network (Schwartz et al., 2015). The study is preliminary but support the feasibility of integrating non-neuronal cells or tissues into brain organoids to enhance neuronal maturation and circuit formation. This is especially significant for investigation of synaptic and circuit dysfunctions that are hallmarks of many neurological diseases.

Encouragingly, biomaterials and bioengineering are providing valuable tools for brain organoid research by introducing molecular, cellular and structural features that normally exist in human brain (Wan, 2016; Yin et al., 2016). The organoids have relied on the self-organizing properties of mammalian cells or bioengineered scaffolds to arrange cells in an organ-like configuration (Lancaster and Knoblich, 2014a; Clevers, 2016; Giandomenico and Lancaster, 2017). While self-organized organoids recapitulate well the early development of organs, bioengineered constructs could consistently produce desired tissue architectures. One study combined these two approaches to create human forebrain tissue by using poly (lactide-co-glycolide) copolymer fiber microfilaments as a floating scaffold to produce elongated EBs, while maintaining the self-organizing capacity of the organoids (Lancaster et al., 2017). These engineered brain organoids show improvement in both neuroectoderm formation and cortical development. Furthermore, reconstituted basement membrane results in the characteristic cortical structure, including spatial organization of polarized cortical plate and radial units. Thus, the bioengineered brain organoids recapitulate the distinctive architecture of cerebral cortex and will be useful for study of cortical development, demonstrating that combination of 3D culture with bioengineering could improve tissue organization and increase reproducibility of brain organoids (Lancaster et al., 2017). It is imperative to combine bioengineering with different organoid approaches to find an ideal combination of techniques that could eventually produce new generation of brain organoids most closely reflecting the human brain (Koch and Ladewig, 2017; Lancaster et al., 2017; Figure 3).

Further improvement of the organoid protocols with the development of technologies, will help to investigate more complicated interactions in brain, such as neuron–glia interactions and neural circuitry. Importantly, it will allow for modeling a larger range of neurological disorders, including disorders of the developing, adult and aging brain, and even phenotypic profiling or therapeutic screening at high-throughput levels (Kelava and Lancaster, 2016a; Quadrato et al., 2016; Di Lullo and Kriegstein, 2017). The successful use of organoids for disease modeling indicates a potential for their application in development of diagnostic and therapeutic approaches or strategies. In terms of personalized medicine, brain organoids generated from patient iPSCs, together with drug testing, diagnostic or therapeutic approaches, would represent a promising direction for organoid technology (Wen et al., 2016; Di Lullo and Kriegstein, 2017; Hartley and Brennand, 2017). Once robust brain organoid protocols are established, a battery of techniques can be applied to probe unsolved issues concerning human brain biology, diseases and therapy. Particularly, in combination with other molecular and cell biology approaches, genome-wide transcriptomic analysis of brain organoids using single-cell RNA sequencing (RNA-Seq) which has been demonstrated as a powerful tool in dissecting cell diversity and defining developmental trajectories of different cell types in both human brain organoids and developing brain (Camp et al., 2015; Quadrato et al., 2017; Xiang et al., 2017; Zhong et al., 2018), are making it possible to deeply characterize human brain organoid models, and to investigate fundamental mechanisms of neurological diseases. Transcriptomic profiling of numerous single cells from patient-derived brain organoids might also help to find novel biomarkers and design personalized treatment strategy (Kelava and Lancaster, 2016a; Di Lullo and Kriegstein, 2017; Zhong et al., 2018). With genome editing approaches such as CRISPR-Cas9, brain organoids can be created to harbor the same genetic defects as patients and further applied to define the function of mutated genes. In addition, patient brain organoids could be genetically repaired; the repaired organoids can serve as isogenic controls and might have the potential for replacement therapy (Muffat et al., 2016a; Li Y. et al., 2017; Seo et al., 2017; Wang et al., 2017). With the development of new automation and other bioengineering techniques, like 3D-printed, scalable set of mini bioreactors named SpinΩ, or bioengineered scaffolds, which allow for reproducible and paralleled production of organoids, patient-derived brain organoids can be grown on a high-throughput scale to test a large set of drugs and find the most beneficial ones for patients (Kelava and Lancaster, 2016a; Qian et al., 2016; Quadrato et al., 2016; Lancaster et al., 2017; Figures 2, 3).

## Conclusion

The failures of potential neurotherapeutics to translate from animal models to patients highlights the need for better predictive preclinical models (Bradshaw and Porteous, 2012; Hagerman et al., 2014; Wang et al., 2015; Wen et al., 2017). Human derived brain organoids are holding the promise for better understanding human brain and diseases than animal models and may help to bridge the gap between model systems and patients (Clevers, 2016; Mason and Price, 2016; Di Lullo and Kriegstein, 2017; Giandomenico and Lancaster, 2017). Although tremendous advances have been made in culture of brain organoids, it is clear that the organoid technologies are not without their disadvantages or limitations. In addition, some ethical issues exist particularly regarding the sources of cells that are used to generate organoids and the potential application of those organoids in humans (Bredenoord et al., 2017; Giandomenico and Lancaster, 2017; Paşca, 2018). While there are still many challenges, we can expect that the organoid research could lead to deeper knowledge of disease mechanisms and personalized treatment for brain disorders in future. It will be exciting to witness how far current brain organoid technology could be pushed forward. Definitely, more efforts from scientists of different sectors, including neuroscience, stem cell biology, neurology, bioengineering and biomaterials, are required to fully realize the potential of brain organoids to model human brain biology and diseases.

## Author Contributions

HW conceived, wrote and revised the manuscript.

## Conflict of Interest Statement

The author declares that the research was conducted in the absence of any commercial or financial relationships that could be construed as a potential conflict of interest.
